# Assisted Reproductive Technologies in Latin America and Europe: a Comparative Analysis of Reported Databases for 2013

**DOI:** 10.1055/s-0039-1693680

**Published:** 2019-08

**Authors:** Oscar Barbosa Duarte-Filho, Paulo Homem de Mello Bianchi, Alexandre Likier Steinberg Lobel, Pedro Felipe Magalhães Peregrino, Carla de Azevedo Piccinato, Sérgio Podgaec

**Affiliations:** 1 Department of Gynecology and Obstetrics, Hospital Israelita Albert Einstein, São Paulo, SP, Brazil; 2 Human Reproduction Center Governador Mario Covas, Clinics Hospital, Faculdade de Medicina, Universidade de São Paulo, São Paulo, SP, Brazil; 3 School of Medicine, Universidade de São Paulo, São Paulo, SP, Brazil

**Keywords:** registries, assisted reproductive technique, in vitro fertilization, intracytoplasmic sperm injection, artificial insemination, registros, técnicas de reprodução assistida, fertilização in vitro, injeção intracitoplasmática de esperma, inseminação artificial

## Abstract

**Objective** To compare the Latin American and European assisted reproductive technology (ART) registries regarding data accessibility and quality, treatment utilization, effectiveness, safety, and quality of services.

**Methods** We performed an ecological study using data from scientific publications of Latin American and European registries that report cycles initiated during 2013 (the most recent registries available until December of 2017). The summarized data are presented as frequencies, percentages, minimum-maximum values, and absolute numbers.

**Results** Reporting clinics and cycle treatments were unevenly distributed between the participating countries for both registries, although access to ART is 15 times greater in Europe. In Latin America, individual services participate voluntarily reporting started cycles until cancellation, birth or miscarriage, while in Europe it varied among countries. It makes the data available from Latin America more uniform, although lesser representative when compared with European ones, given that reporting is compulsory for most countries. The cumulative live birth rate was better in Latin America. Female age, use of intracytoplasmic sperm injection (ICSI), cycles with transfer of ≥ 3 embryos, as well as multiple pregnancy rates were greater in the Latin American Register of Assisted Reproduction (RLA, in the Portuguese acronym). Assisted reproductive technology complications, such as ovarian hyperstimulation syndrome, hemorrhage, and infections were also higher in Latin America, although they are extremely uncommon in both regions.

**Conclusion** Both regions have points to improve in the quality of their reports. Latin America has produced a more uniform reporting, their clinical results are generally comparable and sometimes higher than the European ones. In contrast, the safety of the treatment was higher in Europe, with lower rates of complications, especially multiple pregnancies.

## Abstract (pt)

**Resumo:**

**Objetivo** Comparar os registros das técnicas de reprodução assistida da América Latina e da Europa em relação à qualidade dos dados e acesso, utilização do tratamento, efetividade, segurança e qualidade dos serviços.

**Métodos** Estudo ecológico usando os dados das publicações científicas dos registros da América Latina e da Europa com os ciclos iniciados durante o ano de 2013 (que são os dados mais recentes disponíveis até dezembro de 2017). Os dados são apresentados como porcentagens, valores mínimos e máximos e números absolutos.

**Resultados** Em comum, vemos que o número de clínicas e de ciclos de tratamento varia bastante entre os países dentro de cada região em ambos os registros, embora o acesso às técnicas de reprodução assistida seja 15 vezes maior na Europa. Na América Latina, os serviços reportam voluntariamente os ciclos iniciados até o cancelamento, o nascimento ou aborto, enquanto que na Europa o que é reportado varia entre os países. Isso faz o registro da América Latina mais uniforme, apesar de ser menos representativo quando comparado ao Europeu, dado o caráter compulsório na maioria dos países deste último. A taxa de gravidez cumulativa, idade feminina, uso de injeção intracitoplasmática de espermatozoides (ICSI), ciclos com transferência ≥ 3 embriões e taxa de gestação múltipla foram mais elevados na América Latina. Complicações da reprodução assistida, como síndrome de hiperestimulação ovariana, hemorragia, e infecções também foram mais comuns na América Latina, embora sejam incomuns em ambas as regiões.

**Conclusão** Ambas as regiões têm pontos a melhorar na qualidade dos registros. A América Latina tem produzido um registro mais uniforme, e seus resultados clínicos são comparáveis e, algumas vezes, superiores aos Europeus. Por outro lado, a segurança do tratamento foi maior na Europa, com menores taxas de complicações, especialmente gestações múltiplas.

## Introduction

Determining the most effective treatments for complex medical conditions and the optimal strategies to maximize the quality of delivered care requires detailed, reliable clinical data. Historically, these data have been obtained from randomized clinical trials, but these are expensive experiments to perform and, often, the enrolled study subjects are restricted to a specific population. Conversely, observational studies using data from large national registries provide an opportunity to link current healthcare practices to the respective outcomes, thus improving the quality of care and determining the effectiveness of treatments and health care processes.^1^ ^2^

This scenario is not different than the one observed in the monitoring of the practice of assisted reproductive technologies (ARTs) and of their outcomes. National and international ART registries worldwide have improved over recent years.^2^ The specific goals of centralized ART registries are: to assess interventions by analyzing outcomes, to monitor safety and efficacy trends in national and regional ART centers; to assist infertile couples in the evaluation of costs and benefits; and, finally, to provide reliable data for epidemiological studies and to offer external quality control reports for each center.^3^ ^4^ Developments in information technology, increasing demands for accountability, and stakeholder and government engagement have had a direct impact on this progress, guaranteeing better quality registries in reproductive healthcare.^4^ However, socioeconomic disparities affect the access to ART treatment both in developed and developing countries.^5^

The right to universal access to reproductive health in terms of equal access for equal need has been widely recognized and has been the subject of recent attention by governments.^6^ One of the aspects of this interest is the organization of national quality control registries to meet international standards and accessibility requirements.^7^ The Latin American Registry of Assisted Reproduction (RLA, in the Portuguese acronym) arose as the first multinational centralized registry of this kind, back in 1990.^8^ The European IVF-monitoring Consortium (EIM), under the European Society of Human Reproduction and Embryology (ESHRE), started data collection in 1997. Like its Latin American counterpart, it includes information on *in vitro* fertilization/intracytoplasmatic sperm injection (IVF/ICSI), frozen-thawed embryo transfers (FET), oocyte donation (OD), intrauterine insemination (IUI) and preimplantation genetic diagnosis and screening (PGD/PGS), but also on in vitro maturation (IVM) and frozen oocyte replacements.^9^ The main focus of the present study is to compare data from the two aforementioned selected registries. Due to their similar report format and data processing, the RLA and the EIM provide interesting sources to investigate and compare ART data accessibility, effectiveness, quality, safety, and centralized registries of distinct world regions: Latin America and Europe.

## Methods

### Data Extraction and Quality

Our strategy for the present ecological study was to search scientific publications in Latin America and in Europe that reported data collection for the same time periods: data from ART performed in 2013 in Latin America and Europe (i.e., treatment cycles initiated between January 1 and December 31, 2013), and published by the ESHRE in 2017 and by the RLA in 2016.^8^ ^9^ As only members of the ESHRE and of the RLA can access and use the full registry databases for research purposes, we focused our comparisons in the published, summarized data. No Institutional Review Board approval was therefore required, since neither patients nor center level data were used.

### Statistical Analysis

All of the collected data are descriptive and are presented in table format. Since we have used summarized data from population-wide absolute values, details on how each value was calculated should be looked up in the original publication. The summarized data are presented as frequencies, percentages, minimum-maximum values, and absolute numbers. For comparison between the two reports, we have used statistical hypothesis tests when applicable, and *p* < 0.001 levels were considered as statistically significant. The details of the data collection processes were surveyed to account for differentiate denominator coverage between different databases. This strategy ensured the comparability of data.

## Results

### Extracted Summarized Data

Table 1 presents the characteristics of the RLA and EIM reports on cycle treatments initiated in 2013. Data from RLA are updated yearly and are available online (open access) at the RLA website (www.redlara.com) and published at JBRA Assisted Reproduction and RBMOnline. Data report is voluntary. Privacy and security were ensured at the patient and service (clinics or centers) levels. Individual services participate through a web-based platform, reporting cycles from controlled ovarian stimulation until birth or miscarriage. The RLA gathered data from 158 clinics that belong to 15 of the 20 countries of Latin America, comprising 55,840 treatment cycles (80% of the estimated assisted reproduction treatments performed in the region). Reporting clinics and cycle treatments were unevenly distributed among the participating countries, with Brazil contributing with the greatest number (56 clinics, 24,613 cycles), and Paraguay with the lowest (1 clinic, 39 cycles). The mean access to ART procedures (IVF, ICSI, FET, and OD) was 425 procedures per million women aged between 15 and 45 years old, although a significant variation between countries was also observed (for example, 1,368 per million women in Argentina versus 24 per million women in Paraguay).

**Table 1:** Main characteristics of the reports for the year 2013 from the Latin America Registry of Assisted Reproduction (RLA/REDLARA), and the European IVF-monitoring Consortium (EIM/ESHRE)^8^ ^9^

	Latin America	Europe
**Report / Institution (Abbreviation)** **Report start (years)**	RLA/REDLARA 1990	EIM/ESHRE 1997
**Countries (** ***n*** **)**	20	51
**Countries reporting data (** ***n*** **, % of total)**	15 (75%)	38 (74,5%)
**IVF centers (** ***n*** **)**	NA	1,369
**IVF centers reporting data (** ***n*** **, %)**	158 (NA)	1,169 (85.4%)
**Reporting method**	Cycle-by-cycle	Cycle-by-cycle (8/38) Summary (30/38)
**Compulsory report**	NA	Yes (20/38)
**Timing of cycle reporting**	Retrospective, during the current year	Variable*
**Submission form**	Online, software web-based, by IVF centers	Online, software web-based, by Medical Organization, Health Authority, or personal initiative
**Data validation**	Yes	Yes (16/38)
**Public access to individual clinic data**	NA	Yes (8/38)
**Financial support for registration**	NA	Yes (19/38)
**Access/ per million women between 15 and 45 yearsold**	425	6,210

Abbreviations: IVF, in vitro fertilization; NA, not applicable or not available.

* UK, during the cycle (registered intention to treat)

Similarly, the EIM registry is updated yearly and is available at the ESHRE website (https://www.eshre.eu/Data-collection-and-research/Consortia/EIM/Publications.aspx), and it is published in a scientific journal (Human Reproduction), although full access to the journal requires subscription. The EIM is formed by representatives of each participating country, who are responsible for entering data of their respective national registries in an online form. In 2013, the EIM data record encompassed 38 of the 51 European countries. The reporting of ART is compulsory in 20 countries, and is held by the National Health Authority or by a Medical Organization. It integrates 85.4% of all ART clinics in Europe, and 686,271 treatment cycles were performed in 2013. Differently from the RLA, data from individual clinics are available to the public, but only in 8 countries: Albania, Greece, Montenegro, Romania, Slovenia, Spain, the Netherlands, and the United Kingdom. Overall, the number of ART procedures per million women aged between 15 and 45 years old was 6,210. It is important to note that there was a great variation among the participant countries regarding the proportion of reporting clinics, the size of these clinics, the way data are reported (in 16 countries, the cycles were individually reported, and in 12, a summary was provided), and the access to treatment (14,453 per million women aged between 15 and 45 years old in Denmark, and 1,207 per million women aged between 15 and 45 years old in Moldova). In addition, considering that information on the number of initiated cycles, pregnancy follow-ups and deliveries were missing in some countries, the only complete information regarding outcomes was the clinical pregnancy rate per follicular aspiration. Table 2 summarizes the frequency of use and characteristics of ART from the RLA and the EIM in 2013. Regarding IVF or ICSI, in the RLA, the cycles are based on initiated cycles. In the EIM report, most IVF/ICSI cycles also correspond to initiated ones, but in 7 countries there is only information on cycles that had proceeded to follicular aspiration. There was a ∼ 13-fold difference in the number of cycles reported by the EIM (474,666) as compared with the RLA (36,494). This striking difference persists when analyzing accessibility, that is, the number of procedures relative to the size of the population, since in 2013, Latin America was estimated to have 606 million inhabitants, while there were 740 million people in Europe, according to the Population Reference Bureau (http://www.prb.org/pdf13/2013-population-data-sheet_eng.pdf).

**Table 2:** Frequency of Assisted Reproductive Technologies

	Latin America	Europe	*p-value*
**IVF/ICSI cycles** **(autologous fresh oocytes)**			
Total number of reported cycles*	36,494	474,666	< 0.001^a^
IVF cycles (*n*, %)	5,173 (15.30%)	144,299 (30.40%)	
ICSI cycles (*n*, %)	28,599 (84.70%)	330,367 (69.60%)	
Cancelation rate before follicular aspiration (%)	3.85%	NA	
Follicular aspirations (*n*)	35,089	NA*	
Cancelation rate after follicular aspiration (fertilization failure/no embryo to transfer)	7.24%	NA	
“Freeze-all” cycles	5,168 (14.72%)	NA	
Age distribution of the women			< 0.001^b^
≤ 34 years old	29.22%	44.56%	
35–39 years old	40.13%	36.74%	
≥ 40 years old	30.65%	18.70%	
**Oocyte and/or embryo cryopreservation**			
Cycles for oocyte or embryo cryopreservation (*n*, %)	1,616 (4.42%)	NA	
Number of cycles with thawed oocytes (*n*, %)	NA	6,611 (1.39%)	
**Cycles with donor oocytes or sperm**			
Oocyte donation cycles (*n*, %)	8,434 (23.11%)	40,244 (8.47%)	< 0.001^a^
Cycles with donor sperm (and fresh oocytes) (*n*, %)	NA	17,938 (3.77%)	
**PGD/PGS (fresh and thawing cycles)**			
Cycles with PGD/PGS (*n*,%)	1,920 (3.43%)	10,860 (1.58%)	< 0.001^c^
**Embryo transfers to the uterus**			
Embryo transfers (*n*)	36,009	403,948	
Number of embryos transferred to the uterus (%)^d^			
1	16.90%	31.4%	< 0.001^e^
2	57.20%	56.3%	
3	23.40%	11.5%	
≥ 4	2.50%	1.0%	
Elective single embryo transfers (*n*, %)	512 (2.00%)	NA	
**Intrauterine insemination**			
Cycles with autologous semen (*n*, %)	6,250 (86.65%)	175,467 (80.03%)	< 0.001^a^
Cycles with donor semen (*n*, %)	963 (13.35%)	43,785 (19.97%)	

Abbreviations: ICSI, intracytoplasmic sperm injection; IVF, in vitro fertilization; NA, not applicable or not available; PGD/PGS, preimplantantion genetic diagnosis/screening.

* The total number of cycles refers to the initiated cycles in the RLA report and to the initiated and aspirated cycles in the EIM report – in the majority of European countries, IVF and ICSI cycles refers to the initiated ones, but in 7 of them, there is only information on the aspirated cycles, which are counted together with the initiated ones.

a Proportions of cycles, Latin America versus Europe.

b All age groups, Latin America versus Europe.

c Percentage of total number of PGD/PGS in relation to the total number of cycles in Latin America (*n* = 55,840) and Europe (*n* = 686,271), including frozen cycles, in vitro maturation, and egg donation.

d Sum of European percentages exceeds 100% in the original report, probably due to the approximation in decimals.

e All groups, Latin America versus Europe.

Both reports provide the age distribution of women undergoing ART, and we highlight a statistically significant difference between the regions. As shown in Table 2, in Europe, there was a greater proportion of women ≤ 34 years old treated with IVF/ISCI than in Latin America; conversely, almost a third of women treated with IVF/ISCI in Latin America were ≥40 years old, as compared with ∼ 19% in Europe. Intracytoplasmic sperm injection was more common than IVF in both regions – in Europe, it has been so since 2002–but the proportion of cycles in which ICSI was used was statistically significantly greater in Latin America (84.7%) than in Europe (69.6%). Also, in Latin America, there was a greater proportion of oocyte donation (OD) and of cycles in which PGD/PGS was performed as compared with the European data (*p* < 0.001).^10^ Intrauterine insemination (IUI) was more common in Europe than in Latin America, either in absolute numbers or relative to the number of IVF/ICSI cycles (11.95% versus 24.21%, for RLA and EIM, respectively, *p* < 0.001). In Europe, almost one third of embryo transfer procedures carried just 1 embryo to the uterus (≥ 3 embryos were transferred simultaneously in only 12.5% of cases), which contrasts with Latin America, where 26% of embryo transfers carried ≥ 3 embryos (*p* < 0.001). This reflects in higher multiple pregnancy rates, particularly triplets in Latin America, as seen in Table 3.

**Table 3:** Assisted reproductive technology outcomes in 2013 in Latin America and in Europe

Pregnancy and live birth rates IVF/ICSI		Latin America	Europe	*p-value*
Clinical pregnancy rates – fresh autologous oocytes (per follicular aspiration)	IVF	31.45%	29.6%	0.004
	ICSI	25.75%	27.8%	< 0.001
Clinical pregnancy rates – autologous oocytes (thaw cycles)		33.58%	27.0%	< 0.001
Cumulative live birth rates – autologous oocytes (per follicular aspiration)		28.80%	26.90%	< 0.001
Clinical pregnancy rates – donated oocytes (fresh)		47.25%	49.80%	< 0.001
Clinical pregnancy rates – donated oocytes (thaw)		38.17%	38.50%	0.716
**Delivery rates IUI**				
Autologous semen		14.91%	8.6%	< 0.001
Donor semen		23.36%	11.1%	< 0.001
**Obstetric and perinatal complications (IVF/ICSI)**				
Miscarriage rates (fresh embryo transfers)		18.29%	16.80%	< 0.001
Miscarriage rates (frozen-thawed embryo transfers)		19.52%	19.80%	0.647
Multifetal pregnancies				
Twin pregnancies		20.70%	17.5%	< 0.001
Triplets and more		1.10%	0.5%	< 0.001
**ART complications**				
Severe ovarian hyperstimulation syndrome		0.59%	0.26%	< 0.001
Hemorrhage		0.16%	0.11%	0.0023
Infection		0.05%	0.01%	< 0.001
Maternal mortality (per million)		NA	2.88	

Abbreviations: ICSI, intracytoplasmic sperm injection; IVF, in vitro fertilization; NA, not applicable.

Table 3 also shows clinical outcomes of the cycles. Cumulative live birth rates for autologous cycles are statistically significantly higher in the RLA, while for treatments with fresh donated oocytes, the European results are better. Delivery rates following IUI (partner and donor semen) were higher in the RLA report. Treatment complications (severe ovarian hyperstimulation syndrome, hemorrhage, and infection) were rare in both regions (< 1% of the cycles), but statistically significantly higher in Latin America.

### Unavailable Data for Comparison

Information on cycles cancelled before or after follicular aspiration, cycles performed exclusively for oocyte or embryo cryopreservation, cycles with thawed oocytes, use of donor sperm in IVF/ICSI, gestational carriers, elective single embryo transfers, perinatal and maternal mortality were missing in one or both reports, rendering impossible comparisons.

## Discussion

The present study compared the general characteristics, summarized data and variability among countries from two economically different regions, Latin America and Europe, based on scientific reports of ART regional registries. Data from both registries are updated annually. The delay between the performance of the treatment and the publication of the registries is explained by the time needed until all babies were born and due to data processing.

The higher clinical pregnancy and live birth rates of autologous fresh and thawed IVF cycles in the RLA are possibly the result of a higher number of embryos transferred and to the inexistence of a national single embryo policy in Brazil, the leading country of the region. Miscarriage rates are higher in the RLA than in the EIM, which can be result of a more advanced age of women in the former. In contrast, considering only cycles using donated oocytes (fresh and thawed), a higher clinical pregnancy rate is observed in the EIM.

Interestingly, the data evidenced a higher prevalence of ICSI use over conventional IVF in both regions. The reasons behind this are not fully understood, as ICSI was initially developed as a treatment for male factor infertility (a condition that affects only ∼ 50% of couples seeking treatment), with no evidence of benefit couples without male factor infertility.^11^ ^12^ The more indiscriminate use of ICSI in nonmale factors may explain the lower clinical pregnancy rates in fresh autologous cycles with this technique in Latin America when compared with Europe.^13^

The relationship between the number of IVF and IUI cycles in Europe is 2.16, and in Latin America it is 5. Considering only cases with donor semen use, this ratio rises to 37 in Latin America and to 10 in Europe. These numbers may reflect a greater liberality of IVF indication in Latin America in detriment of IUI, a characteristic inherent in private services that tend to offer the treatment with greater chance of pregnancy per cycle. Thus, IUI cycles is offered to cases with a better reproductive prognosis, which raises the results of Latin America.

Similarities between regions are the heterogeneity among reporting countries, nonuniformity in data submission and, importantly, lack of compulsory reporting by the majority of the countries. The quality of data is dependent on the local regulatory environment, but largely whether data supply at a national level is mandatory or voluntary. Enforced cycle-by-cycle report would promote more robust data gathering, as bias can occur when reporting is not mandatory (the largest clinics and the ones with better results are more prone to report continuously). Furthermore, retrospective data entry allows for the exclusion of cycles cancelled during ovarian stimulation before follicular aspiration, preventing an intention-to-treat analysis.^14^ ^15^ The International Committee Monitoring Assisted Reproductive Technologies provides a Tool Box for ART Data Collection that can be used as a guide for national registries (available at http://www.icmartivf.org/toolbox/toolbox-main.html).

One distinct positive characteristic of the RLA is that it is a single regional registry and not a compilation of national registries, resulting in better data uniformity across countries. Conversely, the EIM compiles information from different national registries, adding another level of complexity, with possible errors in data acquisition. Moreover, the European registry encompasses countries with no mandatory data reporting and also some countries in which reporting is compulsory; this interferes with the reliability or with the relative weight of the data of other nations and with the overall reliability and uniformity of the EIM data.^4^

A major difference between the RLA and EIM data are in the access to ART, which is 15-fold greater in Europe. This is probably due to the socioeconomic disparity between the two continents, as well as to different policies for infertility treatments (government, health insurance companies, or the patient's out of pocket).^16^ We believe that these economic factors lead to undesirable consequences, with disadvantages for Latin America, such as advanced maternal age, higher number of three and four embryos transferred, and less single-embryo transfers and, hence, higher rates of multiple pregnancies. In Australia, patients pay a minor fraction of the cost of ART and frozen embryo cycles.^16^ This relatively supportive environment had led to Australia having one of the highest ART utilization rates in the world.^17^ ^18^ Nevertheless, the scenario will probably improve in Latin America in the near future, since Argentina, Chile and Uruguay have changed their laws in 2013, providing universal access to ART, which will probably result in improved ART quality parameters in upcoming RLA reports.^18^ ^19^

Some countries in the European registry allow public access to the individual results of the clinics, a practice that also occurs in the US report.^20^ ^21^ The RLA does not report these data. Information transparency is a two-sided issue, since it should be viewed with care by patients when choosing the clinic in which they will be treated, as clinics might select out patients considered to have a poor prognosis to improve their overall results.^20^ Furthermore, underreporting of ART adverse events can occur because they are often treated at other institutions (mainly in general hospitals), or even because presenting accurate data may be deterrent to seek treatment, as evidenced by clinics in the United Kingdom that have been recently accused in the press.^22^ One limitation of the present study in comparing the clinical results between the two registries is that we did not have access to the original database used by the two organizations in their publications. Thus, although summarized data work as a clinical reference for regulatory agencies, health professionals and patients, it has some limitations for a more robust scientific evaluation. Even so, some critical observations could be reached presently using the summarized data.

## Conclusion

In conclusion, the continual monitoring of ART practice and outcomes at a uniform, high-quality international level is essential to analyze access to fertility services, treatment effectiveness, and to identify safety issues. Both regions, Europe and Latin America, have points to improve in the quality of their reports. It is important to highlight that the higher pregnancy rates in Latin America, although promising at first glance, are actually of concern regarding the safety of treatment, especially the high rate of multiple pregnancies and complications such as hemorrhage, hyperstimulation syndrome, and infection.
